# Use of baby food products during the complementary feeding period: What factors drive parents' choice of products?

**DOI:** 10.1111/mcn.13689

**Published:** 2024-06-19

**Authors:** Grace Hollinrake, Sophia Komninou, Amy Brown

**Affiliations:** ^1^ Centre for Lactation, Infant Feeding and Translation (LIFT) Swansea University Swansea UK; ^2^ Faculty Medicine, Health and Life Sciences Swansea University Swansea UK

**Keywords:** advertising, baby food, baby food products, complementary feeding, health halo claims, infant feeding, parents

## Abstract

It is recommended that infants are introduced to complementary foods from 6 months old, moving from a solely milk diet to eating a family diet by 12 months old. Although home cooking of family foods is recommended, a rapidly growing market producing baby food products (BFP) such as jars, pouches and snacks has developed. These are often accompanied by marketing claims around nutritional, health and developmental impacts despite research highlighting high sugar content. Although numerous studies have explored drivers of infant formula choice and use, little research has examined the drivers of BFP use. This study used an online survey for United Kingdom parents of infants aged 4–12 months to explore use of BFP alongside perceptions and drivers to purchase products. Overall, 271 parents participated (173 used BFP and 98 did not), with a descriptive analysis of closed items and a thematic analysis for open ended text conducted. The top motivators for using BFP were convenience, time saving, and baby's perceived enjoyment of products. The most purchased puree was fruit based and the most purchased baby snacks were vegetable puffs/sticks, with snack purchases being more common than purees in this sample. Aspects such as perceived healthiness drove choice, with snack foods being seen to enhance self‐feeding skills, appetite regulation and motor development. Those who did not use BFP did not trust them and preferred to feed their baby home cooked foods. The findings are important for professionals working with parents, to support them through the transition to solid foods, particularly around raising awareness of marketing techniques and how to check content of foods to make a more informed choice.

## Key messages

- Parents had multiple reasons for using BFP. The most common motivators were convenience, time saving and infants' perceived enjoyment of them.
- Some parents perceive baby food products to have nutritional benefits due to health halo statements on packaging. Statements such as low in salt or high in fibre led to perceptions that the product was healthy.
- Baby snacks were the most common BFPs used, sometimes by parents who did not use purees as self‐feeding was seen to support development. Almost a quarter offered baby puffs daily compared to one in six using fruit purees daily.

## Graphical abstract

The top motivators for using Baby Food Products were convenience, time saving, and infant's perceived enjoyment of products. The most purchased puree was fruit based and the most purchased baby snack was vegetable puffs/sticks. Baby snack foods were seen to enhance self‐feeding skills, appetite regulation and motor development.

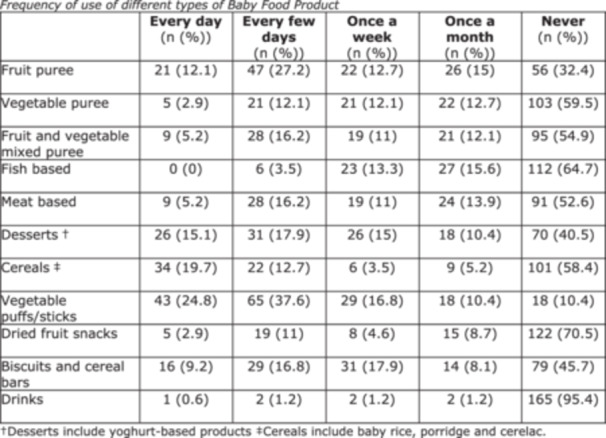

## 1 BACKGROUND

Prevalence of childhood overweight continues to increase with a fifth of children overweight or obese by the time they start primary school (Abarca‐Gómez et al., 2017; Lifestyles Team, NHS England, 2023). Childhood weight often tracks into adulthood where it is associated with a range of comorbidities (Reilly & Kelly, 2011). Alongside weight, childhood undernutrition is an issue in the United Kingdom (UK) with 82% of children not consuming five or more portions of fruit and vegetables a day, increasing risks of hypertension and coronary heart disease (Boeing et al., 2012; Public Health England PHE, 2019).

Establishing healthy eating habits in childhood is recognised as important given eating behaviours learnt early in life are likely to follow into adulthood (Mennella, 2014). However, public health policy often focuses on school‐aged children and misses babies (Department of Health and Social Care, 2020). Given the prevalence of overweight and obesity already established by the time children start school, examining earlier nutrition is key to prevent issues from arising.

Recently, greater attention has been directed towards the first 1000 days of life exploring how infant feeding, including milk feeding and complementary feeding, may impact the development of food preferences and eating behaviours (Hoffman et al., 2019). Although research is growing in understanding how introduction to solid foods may influence infant weight (Moorcroft et al., 2011; Pearce et al., 2013; Townsend & Pitchford, 2012; Williams Erickson et al., 2018) and dietary intake (Pearce & Langley‐Evans, 2022; Rowan et al., 2019, 2022), most research exploring the first years of life focuses on milk feeding rather than the complementary feeding period.

Baby food products (BFP), such as purees and baby snacks, are foods marketed as suitable for complementary feeding. Since the introduction of these products in the 1930s, their use has become widespread and synonymous with introducing solid foods to babies (Bentley, 2014). In the UK, the BFP industry has grown over the last 5 years and is predicted to continuously increase over the next 5 years also, with revenue estimated to have reached £960 million, and expected to reach £1.1 billion by 2028 (Bedford, 2023). However, concerns have been raised around the sugar content of some foods. The sugar content, particularly for fruit and vegetable purees is higher than recommended, with fine processing of these products leading to very high 'free sugar' content (Garcia et al., 2020a). Processing strips out the fibre from these foods, leaving sugar to be easily absorbed, with a higher free sugar intake associated with overweight and tooth decay in older children (Childs & Sibson, 2023). Likewise, snack foods such as dried fruit products, rice cakes and biscuits, due to the free sugar content have as much as 61 g per 100 g, far above the recommended 5 g per 100 g total sugars threshold for low sugar products (Garcia et al., 2020a; NHS, 2022) Research looking at BFP across Europe found even savoury products had high sugar composition due to the addition of fruit purees, most seen in the UK (Hutchinson et al., 2020). There are growing concerns about the health impacts of UPF, the degree of which they make up infant diet, and how they are promoted as healthy foods for infants.

Whilst research highlighting the scale, sophistication, and impact of formula milk marketing upon purchasing is clear, less attention has been paid to marketing of baby food products, but similarities may arise. For example, formula companies use slogans promoting health and developmental claims, leading to purchasing of higher priced milks despite many claims being unsubstantiated (Brown et al., 2020; Munblit et al., 2020). BFP manufacturers use similar slogans on products, but little research has considered how these are interpreted by parents.

Two qualitative studies in the UK, with parents of 4–7 month old infants found BFP were seen as more convenient, both in and outside the home, than homemade food with some perceiving them as superior and safer than foods they could make their infant (Isaacs et al., 2022; Maslin et al., 2015). Other influences included taste, perceived healthiness, and cost effectiveness (Isaacs et al., 2022; Maslin et al., 2015). Furthermore, mothers' opinions on BFP were influenced by parity, previous experience of weaning, and level of education (Maslin et al., 2015).

Based on the rise of different BFP and marketing strategies to parents and the potential implications this might have for infant diet and later health, this study aimed to explore the perceptions of BFP amongst UK parents of infants aged 4–12 months, who had been introduced solid foods. An online questionnaire was used to examine how frequently parents are using BFP, motivators for BFP general use, and specific product use.

## 2 METHODS

### 2.1 Definitions

The following definitions are used in this study:

- *Baby food products [BFP]*—an umbrella term meaning all commercially available purees and baby snacks advertised for babies aged 4–12 months. It does not include baby stock cubes, make at home kits or anything advertised for over 1 years old.
- *Baby snacks* – an umbrella term meaning dried fruit‐based products, vegetable sticks/puffs, cereals, biscuits and cereal bars, and drinks advertised for babies aged 4–12 months.

**Design:** A self‐report online survey aimed at parents of a baby aged 4–12 months who had been introduced to solid foods.

### 2.2 Participants

Participants were UK parents, over 18 years old, of infants aged 4–12 months, who had been introduced to solid foods. Exclusion criteria included not being able to complete the questionnaire in English or a lack of capability and capacity to give informed consent. Although the National Health Service recommend introducing solids from 6 months old (NHS, 2020), we chose the age range of 4–12 months as some BFP are marketed from 4 months and many parents still introduce solids before the 6‐month guidelines. BFP are more likely to be introduced to infants who receive solid foods before 6 months old (Brown & Lee, 2010).

Ethical approval was obtained from a University Research Ethics Committee. Ethical approval was granted by the Research Ethics Committee at the College of Human and Health Sciences, Swansea University. All aspects of the Declaration of Helsinki were followed. All participants gave informed consent before participating.

### 2.3 Measures

All data were collected through a self‐report online questionnaire (See Appendix 1) hosted by Qualtrics.

The questionnaire comprised of open and closed questions and examined:

- Participant demographic (parent age, sex, education, marital status, employment status, ethnicity, parity)
- Infant age and sex
- If baby food products had ever been used

Participants who had given BFP to their child completed further questions regarding:

- Reasons for using BFP [Response options via a 5‐point Likert scale strongly agree‐strongly disagree].
- Use of different BFP Brands and types [Response options via a 6‐point Likert scale from never used to use more than once a day]
- Reasons for using certain brands and types [Response options via a 5‐point Likert scale strongly agree‐strongly disagree]

Open ended boxes were used to expand responses relevant to motivations for using BFP and specific BFP products.

Participants who had never given BFP completed one separate question giving their reasoning for not using BFP in an open‐ended answer box.

Themes for the reasons for using BFP were built from existing literature around reasons for introducing solid foods and drivers of BFP (Brown, & Rowan, 2016) and extrapolated from literature examining formula milk advertising (Brown et al., 2020; Rollins et al., 2023).

### 2.4 Procedure

Data were collected from May to July 2021, for 8 weeks. The online questionnaire was created using online survey software by Qualtrics UK and adverts shared on social media. Posts were shared on the academic pages of the research team including Facebook pages and Twitter with encouragement for interested viewers and organisations to share further. During the study period the advert was shared over 200 times across social media platforms (with further sharing that could not be tracked for privacy reasons), with a post reach of at least 90,000 accounts.

Adverts contained brief details of the study and inclusion criteria. Interested parents could click the link and were directed to the participant information sheet and consent form. Once the questionnaire was completed a debrief statement was given, thanking them for participation, stating if they had any questions about the research to contact research team, and if they had any concerns about feeding their baby to contact their health visitor or the NHS website.

### 2.5 Data analysis

Statistical analysis of quantitative data were conducted using SPSS version 26, generating descriptive statistics. Participant responses were considered complete if full demographic data and infant age was provided and over 90% of main questions completed. For each Likert scale the percentage who strongly agreed and agreed was generated for each statement.

For the open‐ended boxes, thematic analysis of qualitative data, was conducted using paper, coloured pens, and Microsoft Excel. Braun and Clarke's (2022) six step approach was used, with the first author familiarising themselves with the data, generating initial codes, searching for then reviewing themes, defining and naming themes and then writing the report. To enhance trustworthiness of the data (Lincoln & Guba, 1986), initial coding was completed by the first author, then discussion with the last author reviewing proposed themes and subthemes.

## 3 RESULTS

Two hundred and seventy‐one parents' questionnaires were eligible for analysis. Full parent demographic data is shown in Table 1. Two hundred and four (75.3%) parents had degree level of education or higher, and 250 (92.3%) parents were White/White British ethnicity. Infant mean age was 8.5 months (range 4–12 months, SD = 2.08), with 126 females and 137 males. One hundred and seventy‐three (63.8%) parents had used BFP and 98 (36.2%) had never used BFP.

**Table 1:** Participant demographic data.

Demographic	Frequency	Percentage
Age (years)		
20–29	76	28.0
30–39	174	64.2
>40	21	7.7
Sex		
Female	269	99.3
Male	1	0.4
Education level		
No formal qualification	2	0.7
GCSE or equivalent	12	4.4
A Level or equivalent	53	19.6
Degree level or equivalent	104	38.4
Postgraduate or equivalent	100	36.9
Marital status		
Married	177	65.3
Cohabiting	78	28.8
Divorced	1	0.4
Single	13	4.8
Employment status		
Working, full‐time	96	35.4
Working, part‐time	91	33.6
Unemployed	84	31.0
Ethnicity		
White/White British	250	92.3
Asian or Asian British: Pakistani	1	0.4
Asian or Asian British: Indian	2	0.7
Asian or Asian British: Other	1	0.4
Mixed or multiple	10	3.7
Irish	4	1.5
Other	2	0.7
Prefer not to say	1	0.4
Parity		
One	119	43.9
Two or more	150	55.4

### 3.1 Introduction of solids

Parents gave the age (in weeks) infants were first introduced to solids. The mean age solids were introduced was 24.44 weeks (SD: 2.68) with a median age of 25 weeks.

### 3.2 Reasons for using BFP

Parents indicated how strongly they agreed with a series of reasons for using BFP. The main motivators for using BFP were convenience, time saving, safe to feed baby, and the perception that their baby likes them (Table 2). BFP being a healthier choice was not a strongly agreed with nor disagreed with statement, most parents voted indifferently. Of those with more than one child, 73% (*n* = 72) agreed they used BFP as they used them with older siblings. Moreover, 93% (*n* = 68) of parents with one child agreed this is the reason they used BFP. Overall, parents agreed with a mean 6.5 (SD: 2.41) statements, with a range from 2 to 13 showing the complexity of influencing factors.

**Table 2:** Reasons for using BFP.

Reasons	Strongly agree/Agree	
	*n*	%
Convenient	164	94.8
Saves time	152	87.9
My baby likes them	133	76.9
Safe to feed my baby	129	74.6
Good way to introduce new tastes and textures	88	50.8
Friends with babies use BFP	79	45.6
Used with my other children	72	41.6
This is my first baby	68	39.3
BFP show what is suitable to feed at specific ages	59	34.1
Healthcare professional recommended	52	40.1
Affordable	50	28.9
Given money off coupons	40	23.1
Family member recommended	24	13.9
Saw promoted on social media	21	12.1
Healthier choice	17	9.8
Saw promoted in a baby magazine	16	9.2

### 3.3 BFP types

Parents were asked how frequently they used different types of BFP (Table 3). For puree products, fruit was most likely to be offered every day. For snacks, vegetable sticks/puffs were most likely to be offered every day. Examining the most commonly offered daily BFP, vegetable sticks/puffs were offered every day by 24.8% (*n* = 43) of the sample, cereals by 19.7% (*n* = 34) and desserts by 15.1% (*n* = 26). Notably fruit purees were the fourth most frequently used product at 12.1% (*n* = 21). Other products were rarely used daily. Not everyone used BFP daily, 45% (*n* = 77) of parents said they use something daily or more.

**Table 3:** Frequency of use of different types of BFP.

	Every day (*n* (%))	Every few days (*n* (%))	Once a week (*n* (%))	Once a month (*n* (%))	Never (*n* (%))
Fruit puree	21 (12.1)	47 (27.2)	22 (12.7)	26 (15)	56 (32.4)
Vegetable puree	5 (2.9)	21 (12.1)	21 (12.1)	22 (12.7)	103 (59.5)
Fruit and vegetable mixed puree	9 (5.2)	28 (16.2)	19 (11)	21 (12.1)	95 (54.9)
Fish based	0 (0)	6 (3.5)	23 (13.3)	27 (15.6)	112 (64.7)
Meat based	9 (5.2)	28 (16.2)	19 (11)	24 (13.9)	91 (52.6)
Desserts^a^	26 (15.1)	31 (17.9)	26 (15)	18 (10.4)	70 (40.5)
Cereals^b^	34 (19.7)	22 (12.7)	6 (3.5)	9 (5.2)	101 (58.4)
Vegetable puffs/sticks	43 (24.8)	65 (37.6)	29 (16.8)	18 (10.4)	18 (10.4)
Dried fruit snacks	5 (2.9)	19 (11)	8 (4.6)	15 (8.7)	122 (70.5)
Biscuits and cereal bars	16 (9.2)	29 (16.8)	31 (17.9)	14 (8.1)	79 (45.7)
Drinks	1 (0.6)	2 (1.2)	2 (1.2)	2 (1.2)	165 (95.4)

^a^ Desserts include yoghurt‐based products.

^b^ Cereals include baby rice, porridge and cerelac.

### 3.4 Motivations for using purees

Parents were asked via an open‐ended question why they used purees. Three main themes were identified – versatility, enjoyment, and convenience.

#### 3.4.1 Versatility

This theme illustrated how purees were used. Parents often stated how a puree could be combined with other foods or used for self‐feeding, rather than talking about simply directly feeding from the jar. e.g., parents described combining fruit purees with other foods to add flavour such as using it on toast or in porridge.

*“For use in yogurt/porridge/on toast”* (Mother aged 29, baby 6 months)

*“Convenient for mixing into porridge or onto pancakes.”* (Mother aged 33, baby 8 months)

The ability for the infant to suck directly from the pouch was also often mentioned.

*“Baby can suck straight from the pouch”* (Mother aged 39, baby 10 months)

*“Fruit pouches which baby can suck herself as dessert or on the go”* (Mother aged 25, baby 11 months)

#### 3.4.2 Enjoyment

Babies were often perceived to really enjoy the flavours of purees, with parents buying the products they believed their baby would enjoy.

*“Baby loves the flavours”* (Mother aged 28, baby 8 months)

*“Little ones favourites”* (Mother aged 23, baby 8 months)

#### 3.4.3 Convenience

Purees were seen as convenient, especially when outside of the home. They were also used if adults were consuming a different meal.

*“Don't need to be refrigerated so are easy to keep in a snack bag”* (Mother aged 35, baby 12 months).

*“To feed our baby if for example we as a family have a takeaway which isn't suitable to feed our baby”* (Mother aged 30, baby aged 8 months).

Within this, the convenience of being able to offer their baby new tastes and textures at little cost or preparation time was valued.

*“As my baby grows I like to try new flavours but don't want the costs and time involved in buying all the ingredients just to have him refuse to eat it. Pouches are an inexpensive and easy way to see what flavours/textures he likes without all of the time.”* (Mother aged 35, baby 9 months)

### 3.5 Motivations for using baby snacks

Motivators for using baby snacks were more complex. Although some similar themes to purees were identified, additional reasons such as distraction or developing feeding skills also arose. These snacks were often seen as safe, nutritious, and helping baby's development. Five themes were identified.

#### 3.5.1 Convenience

Baby snacks were again seen as a convenient food to be used outside the home, often in part because they were seen as less messy than other foods.

*“Handy on the go”* (Mother aged 39, baby 10 months)

*“They don't make her hands sticky or messy”* (Mother aged 29, baby 11 months)

#### 3.5.2 Distraction

Parents also used snacks to distract an infant when they were in the pushchair or unsettled. Sometimes snacks were used until other foods, or a breastfeed could be given.

*“Used mostly for distraction in buggy”* (Mother aged 39, baby 10 months)

*“I can give her them to eat in the car seat if she's upset when I'm driving”* (Mother aged 32, baby 8 months)

*“To eat whilst I'm cooking otherwise she cries for food.”* (Mother aged 29, baby 10 months)

#### 3.5.3 Perceived enjoyment

Parents often perceived their baby to really enjoy snacks with some rating them as their baby's favourite foods. Sometimes parents perceived their baby as liking a particular brand or product the most. Others limited snacks using them as a treat, driven by how much their baby enjoyed them.

*“Because they are snacks she wants and likes to eat.”* (Mother aged 24, baby 10 months)

*“As a treat, baby enjoys them.”* (Mother aged 32, baby 10 months)

#### 3.5.4 Skill development

Snacks were often seen as a way to encourage babies to develop self‐feeding skills. They were seen as being easy to chew and hold, enabling baby to feed themselves which was seen as a positive step.

*“To allow him to practice independent eating”* (Mother aged 30, baby 8 months)

“easy melty finger food for her” (Mother aged 26, baby 11 months)

*“Suitable for his level of dexterity and chewing ability…Support self‐feeding.”* (Mother 38, baby 6 months)

Within this certain baby snack foods were chosen as they were seen as a safe way to encourage self‐feeding.

*“I feel they are safer to eat.”* (Mother aged 31, baby 12 months)

#### 3.5.5 Perceived as healthy

Parents bought products due to health reasons including allergies and perceived low salt and sugar content. Some compared baby snacks to snacks they give older siblings perceiving baby snacks as a healthier alternative.

*“Can't see they do much harm as mainly corn ‐ no sugar/salt etc”* (Mother aged 35, baby 8 months)

*“Melty puff style things. I don't want to give him crisps so give a few of these as an alternative.”* (Mother aged 27, baby 11 months)

*“Baby Biscotti, as an alternative to biscuits when baby is with siblings.”* (Mother aged 35, baby 12 months)

### 3.6 Reasons for using certain brands or products

Parents then rated their motivators for using specific brands and products (Table 4). The most common reasons were, ‘*My baby likes them’*, ‘*The product has nutritious/natural/real ingredients’*, and ‘T*he product is full of fruit and vegetables’*. Parents agreed they buy certain brands and products because they are affordable, not because they are most expensive. This shows the sample is more concerned with the affordability of products, and potentially parents perceive expensive as not necessarily healthier.

**Table 4:** Reasons for choosing specific brands and products.

Reasons	Strongly agree/Agree	
	*n*	%
My baby likes them	163	94.2
They include nutritious/natural/real ingredients	162	93.6
They are full of fruit and vegetables	148	85.5
I trust this brand	134	77.5
It is a price I can afford	125	72.3
It is a brand/type I can always buy easily	112	64.7
It is sold in my local shop	110	63.6
It is best quality	110	63.6
Organic ingredients	93	53.8
They are cost‐effective compared to other brands	83	48
It contains a variety of ingredients	80	46.2
It is the same I fed my other children (if applicable)	64	37
Other people I know recommended them	50	28.9
The packaging is environmentally friendly	50	28.9
They include lots of different ingredients	38	22
Vegetarian composition	34	19.7
It is gluten‐free	6	3.5
It is most expensive	4	2.3
Halal certified	3	1.7

Parents were then asked if there were any other factors they considered when buying certain brands and products. Five main themes were identified.

#### 3.6.1 Perceived healthy or suitable ingredients

Ingredients in the product were frequently mentioned. This included products that were lower in salt or sugar and had fewer ingredients overall.

*“[Don't] contain lots of fruit ‐ I don't like to feed her too sweet or high in sugar from the fruit.”* (Mother aged 32, baby 8 months)

“*Usually if it has only a few ingredients and no fillers I would be more likely to buy it.*” (Mother aged 36, baby 8 months)

Within this, additional dietary requirements were mentioned. Commonly this was for dairy intolerance, but veganism was also discussed.

*“That it is milk free and that allergens are easy to see.”* (Mother aged 30, baby 10 months)

*“It's healthy and vegan.”* (Mother aged 23, baby 10 months)

A core part of this theme was also what perceived ‘healthy’ ingredients were added to or labelled as high in the food. Health halo statements were a big draw particularly for phrases such as organic, natural ingredients and additives.

*“Any ‘healthy’ related terminology”* (Mother aged 31, baby 12 months)

*“Unnatural ingredients, if its something you cant make at home then we try to avoid it as a product.”* (Mother aged 35, baby 12 months)

*“No additives, organic”* (Mother aged 37, baby 6 months)

However, some mothers spoke of how these statements decreased their likelihood of purchasing, highlighting potentially a deeper understanding of health halo statements.

*“I [don't] like when they [claim] to be all natural etc because realistically they aren't.”* (Mother aged 31, baby 10 months)

*“Organic ‐ think the word is over used does not increase likelihood.”* (Mother aged 25, baby 7 months)

#### 3.6.2 Avoiding certain brands

Some mothers in the survey stated that they specifically avoided certain brands based on their perceived behaviour. If a brand advertised the product to be suitable from 4 months (as against the recommended 6 months+) or was produced by a company that made breastmilk substitutes, these products were avoided.

*“Avoid anything to do with formula companies or their branding.”* (Mother aged 31, baby 7 months)

*“Advertised as 4* + , *as I don't like to feed into the idea that early weaning is ideal.”* (Mother aged 20, baby 10 months)

#### 3.6.3 Packaging and environmental reasons

Different aspects of packaging were mentioned. Some appreciated clear labelling, which companies should aim to have already, this implies some packages are not clear.

*“Clear labelling”* (Mother aged 40, baby 10 months)

Some parents also chose or avoided certain products due to their packaging. Recyclable materials were preferred by some, but also were seen as sometimes less practical outside of the home.

*“I avoid glass jars. I prefer pouches or plastic for use when I am away from home as I find them more practical.”* (Mother aged 37, baby 9 months)

*“I hate pouches, hard to use and not environmentally friendly.”* (Mother aged 38, baby 9 months)

#### 3.6.4 Storage and waste

Certain products were purchased because of factors such as size, storage and whether they were ‘ready to go’ and needed little preparation time.

*“Size of packet ‐ will it go to waste if not finished?… Storage ‐ does it need to be kept refrigerated etc.”* (Mother aged 39, baby 10 months)

*“Long shelf life for snacks so an open bag can last a while”* (Mother aged 37, baby 6 months)

#### 3.6.5 Products that suited a baby‐led weaning approach

Although baby led weaning is often associated with home cooking, some parents specifically chose products because they were seen as finger foods that would fit with a baby led weaning approach. Snack products were seen to support self‐feeding, or for use by others if anxieties around choking were high.

*“Puff type snacks, used as a ‘vessel’ for homemade mushed foods spread onto them for baby to do baby led weaning.”* (Mother aged 33, baby 8 months)

*“Wafers and puffs ‐ a convenient snack when out or with grandparents who aren't confident with blw”* (Mother aged 23, baby 6 months)

However, following a baby led approach did appear to limit the types of products that were used. Purees tended to be avoided by this group, or foods that could easily be cooked at home.

*“We do mostly blw so not many sloppy food.”* (Mother aged 35, baby 7 months)

*“I avoid carrot. Everything seems carrot based and as I do baby led weaning carrots are good for me to cook at home as sticks.”* (Mother aged 29, baby 7 months)

### 3.7 Why do parents not use BFP?

Parents who had never used BFP were asked why they did not purchase them. Themes included homemade food, price, health and learning, not the right time, and distrust.

#### 3.7.1 Homemade food

This was the most common reason given by parents. Many alluded to the idea BFP are ‘*unnecessary*’ or ‘*not needed*’ as they can make their own purees or give whole foods. Many parents spoke of how they were doing baby‐led weaning and so BFP were ‘*not appropriate*’ or ‘*don't fit*’ with this complementary feeding approach.

Parents explained babies can eat the same food as adults, so their baby eats the same as the rest of the family, a given benefit of this is inclusion in family mealtimes. Others explained they do not buy ready meals for the family so do not for their infant. One parent described not using BFP due to *‘laziness’* (Mother aged 34, baby 4 months), which would usually not be associate with feeding baby's homemade meals.

*“Easier to include baby in family foods and mealtimes”* (Mother aged 35, baby 8 months)

*“Don't tend to buy shop bought meals for me and my partner so why give them to the baby.”* (Mother aged 39, baby 8 months)

#### 3.7.2 Price

Many parents mentioned in their response BFP are expensive or making their own food for their baby is cheaper.

*“They [BFP] are expensive.”* (Mother, aged 31, baby 9 months)

“*Prefer making my own as cost effective.*” (Mother, aged 27, baby 6 months)

“*To me baby products are a waste of money.*” (Mother, aged 26, baby 7 months)

#### 3.7.3 Health and learning

Many parents believed BFP were not as healthy as homemade foods as they contain high levels of sugar, salt, and additives, and because they are not as fresh or natural. This is interesting as it shows an understanding of BFP content and goes against the marketing.

*“I'm also [conscious] of what baby food products contain; they are often highly processed, some contain a lot of sugar and salt and even the ‘healthy’ ones often add fruit to savoury dishes to make it sweeter which I don't think is good for babies.”* (Mother aged 33, baby 7 months)

Some parents mentioned babies needing to learn from these early eating experiences, from the tastes and textures, to having a positive attitude about food. This highlights these parents knew about the importance of experiences during complementary feeding.

*“I want my baby to be able to taste the [individual] foods…Trying to encourage a positive attitude with food.”* (Mother aged 39, baby 8 months)

#### 3.7.4 Not the right time

As baby is too young or they are at the start of complementary feeding was the reason for not using BFP some parents. More specifically that baby is too young for snacks, a comment which follows complementary feeding guidance, but indicates baby snacks may be purchased in the future.

*“Too young for me personally at present”* (Mother aged 40, baby 6 months)

*“We don't offer snacks (currently)”* (Mother aged 26, baby 7 months)

#### 3.7.5 Distrust

A theme running throughout comments was the lack of trust in BFP, whether they personally or knew acquaintances who had experienced a negative event involving BFP. This is interesting as BFP are regulated and approved to be sold, but there is this distrust around feeding them to infants.

*“With my second son 14 years ago, i gave him a shop bought jar and there was a piece of plastic with it and he [choked] on it.”* (Mother aged 34, baby 6 months)

*“Don't trust ingredients and additives.”* (Mother aged 41, baby 6 months)

## 4 DISCUSSION

This research aimed to explore parents' motivators for using, and perceptions of BFP. Although research has explored drivers for different formula milk use (Brown et al., 2020), reasons for using different commercial BFP over home‐cooked family foods has received less attention. The global baby food market is growing, with increased advertising revenue (Bedford, 2023; Garcia et al.,  2020a). Given increasing concern around the nutritional content and value of such products (Bridge et al., 2021; Hutchinson et al., 2020; Westland & Crawley, 2018) it was important to understand parents' perceptions and motivators for use. Our survey found a wide range of reasons for using BFP, some based around lifestyle and convenience, but others driven by potential misperceptions of the nutritional value and need for such products. However, it must be noted, our sample is relatively small and skewed towards white, older mothers with a higher level of education. Further research is needed to explore larger, more representative groups motivators for using BFP or not. The findings are important for those working to support parents through the transition to solid foods.

Limited previous research has examined reasons for BFP use finding parents typically choose products based on convenience, infant enjoyment of products, and perceived safety over home cooking (Isaacs et al., 2022; Maslin et al., 2015). Our data expands on these reasons, in a larger sample, highlighting more current reasons for use. The BFP market has grown rapidly over the last decade with multiple new products and marketing practices (Crawley & Westland, 2017; Garcia et al.,  2020b), and our findings highlight the need for greater awareness around infant nutrition choices and changes in legislation on BFP marketing and content so that parents can trust the products they purchase.

One of the most common drivers of BFP use was perceived enjoyment of the product by infants. This is likely to be based on infant cues such as increased consumption or smiling during feeding (Forestell & Mennella, 2017; Mennella, 2014). Research exploring content of common BFP has found a large proportion have high sugar content, often based on apple or pear ingredients meaning they are sweeter tasting (Brunacci et al., 2023; Garcia et al., 2016; Thorisdottir et al., 2024). Additionally, these are free sugars, due to the processing the fruit has gone through, meaning the sugar is more available and can negatively impact oral health (Childs & Sibson, 2023). Infants are naturally predisposed to like sweet tasting foods (Mennella, 2014). Parents are often anxious their baby will consume enough solid foods, especially at the start, and may continue to purchase products they perceive their baby likes due to this.

Indeed, the most used puree in our sample was fruit. This raises concerns, due to increased consumption of sugar, potential over consumption of energy, due to palatability, and the potential for infants to eat a diet with little variability (Westland & Crawley, 2018). It is also likely that if infants become accustomed to sweet tasting foods, they may be less likely to accept more bitter tasting vegetables. Research has shown infants can naturally be hesitant to try new foods, especially those with bitter tastes (Mennella, 2014). Parents might need to offer these foods up to 8–15 times (Ventura & Worobey, 2013). If infants are showing preference for more sweeter tasting products, this could potentially impact upon longer term food acceptance (Caton et al., 2014).

Additionally, there is often a mismatch between the name of a product, its contents, and its taste. Many purees, especially those labelled 4–8 months, are apple or pear based regardless of their name (Garcia et al., 2016). There are currently no regulations in the UK on ingredient percentage in product and using that ingredient in the name, therefore, the name of the product may not reflect the ingredients list. Research investigating Australian pouches found only two products out of nearly 300 were nutritionally adequate, meeting WHO and Australian complementary feeding guidelines, and stated the marketing claims and labelling are misleading (Brunacci et al., 2023; World Health Organisation, 2023). Some parents in our sample were using specific products to introduce new tastes and textures but it is unlikely that the flavour experience matches the name of the product and certainly not to the same extent that the 'whole' food item such as a mashed blueberry would.

There is additional concern around the smooth textures of purees, particularly those aimed at younger babies. Learning to chew and manipulate different foods is an important part of the learning experience (Simione et al., 2018). Very smooth purees however are easily consumed which may lead to a perceived or real preference of enjoyment by the infant, encouraging further use.

Convenience was another common reason for use. Mostly, the convenience of BFP for use away from home, spanning from on the school run to a day out to on holiday. Our survey found parents want to ensure they have food to hand wherever they are, which is easy to give to baby to self‐feed and without mess. However, playing with food, creating mess, and exploring different textures and tastes is an important part of learning to eat and enjoy a range of foods (Coulthard & Sealy, 2017; Dazeley & Houston‐Price, 2015). Many of the commercial snacks offered were viewed as positive because they avoided this.

Baby food pouches were seen as particularly convenient as parents offered the pouch to the infant to squeeze and suck the puree out, although this is not recommended by many manufacturers. This practice can be concerning as if done frequently it does not offer an optimal learning experience. The food is not viewed meaning the infant does not get a feel for its smell or appearance and the amount consumed cannot be accurately viewed potentially leading to over consumption (Crawley & Westland, 2017). Sucking food from a pouch also uses different muscles, whereas chewing is important in helping to develop dental and mouth musculature formation (Simione et al., 2018).

Our findings also showed a significant proportion of parents were offering their baby snacks. A quarter offered these daily, with some parents in this group not being typical BFP users and not offering purees. This area of BFP has rapidly increased in recent years with a variety of products described as helping to support development or nutritional needs, in the UK and other countries (Garcia et al.,  2020a; Thorisdottir et al., 2024). Amongst our sample vegetable puffs/sticks were the most common product used. Alongside convenience and perceived enjoyment, parents felt these products helped to introduce new textures and to support self‐feeding. They expressed feeling comfortable with these due to the melt in the mouth characteristic (Moore et al., 2019). Some perceived these products as a healthier version of snacks for older siblings especially if health halo statements around fruit, vegetable, or fibre content were added.

Although self‐feeding may help to support infant fine motor skill development and encourage enjoyment of foods (Brown et al., 2017) and finger foods are recommended from the start of complementary feeding by the NHS (NHS, 2020), these foods are unlikely to positively meet those goals. Texture wise these snacks lack fibre and easily dissolve meaning little chewing is needed. Frequent snacking may also lead to lower consumption of more nutrient dense foods or breast or formula milk which should remain the predominant part of an infant's diet through the introduction of complementary foods (World Health Organisation, 2023).

The nutritional content of these snacks labelled as ‘vegetable’ is misleading. Health halo statements are a growing concern for BFP. Research has shown adding ‘fruit’ to a sugary product increases perception that it is a healthy option (Sütterlin & Siegrist, 2015). Additionally, many infant snack foods have high levels of free sugars (Sparks & Crawley, 2018). Free sugars are not recommended at all for children under 24 months and the World Health Organisation has drawn attention to their growing content in BFP (World Health Organisation, 2023). The labelling of such snacks with health halo statements such as ‘no added sugar’ is concerning. Research in Australia found claims of ‘no added sugar’ on product packaging, despite the addition of free sugars (Brunacci et al., 2023). Snack intake during infancy can contribute to exceeding recommended energy and fat intake and may be contributing to childhood higher weight (Loughrill et al., 2017; Zand et al., 2015).

Snacks were also often used to distract infants, when out or about or when upset. This is potentially problematic if foods are over consumed but may also play a role in the development of emotional eating. We know from research with older children that using food to soothe emotions or to distract can impact upon emotional eating in later childhood (Steinsbekk et al., 2018). A large cohort study found infants who are emotionally fed were of higher weight at 6 and 10 years old; this was associated with the child emotional eating (Jansen et al., 2019). Therefore, although there is limited research, emotional feeding or using snacks to distract from negative emotions may need to be avoided as it may be learnt and follow on into childhood and adulthood, with increased risk of overweight.

We also explored why some parents did not use BFP. From our findings it is clear some parents are aware of the nutritional content of BFP, which dissuaded use. These parents typically offered their baby home cooked or family meals instead, with some eating with their baby. These choices will be beneficial for later eating behaviours as parents are role modelling behaviours from a young age (Finnane et al., 2017; Scaglioni et al., 2018). This raises important questions as to whether increased awareness of nutritional content and health halo statements may decrease reliance on BFP. However, it is likely broader social factors may be driving use, such as time demands, maternal mental health, and the cost‐of‐living crisis.

Baby led weaning (BLW) emerged as a reason for low or no BFP use. By its nature this approach encourages infants to self‐feed family foods and use of BFP amongst families who follow BLW is typically lower than those who choose a spoon‐feeding approach (Rowan et al., 2019, 2022). Research examining outcomes of BLW often finds infants who follow this approach are less likely to be rated as fussy eaters later in toddlerhood although impact on weight gain trajectories is more mixed (Brown et al., 2017). It is difficult to adopt a diet high in BFP when choosing to follow BLW, but manufacturers appear to have adapted to this self‐feeding approach by increasing range of finger food snack products (Garcia et al., 2020b). Indeed, a subgroup of parents in our survey as described above used snacks as part of a BLW approach even though they did not use purees. It is important to ensure parents are aware not all finger foods hold nutritional value and although self‐feeding likely supports motor development, nutritional content matters too.

Notably snacks such as vegetable puffs were sometimes seen as a safe way to introduce self‐feeding, or for family members who were unsure of BLW to use. Fear of choking is common (Brown, & Lee,  2013; Cameron et al., 2012; Graf et al., 2022) but infants at 6 months old are developmentally ready to self‐feed and overall choking likelihood is low, and not higher amongst infants who self‐feed (Brown, 2018). Marketing may be playing on these fears by producing soft or ‘melty’ foods with little nutritional value that are unlikely to teach infants the same self‐feeding skills as textured whole foods.

The research does have its limitations. As with a lot of infant feeding research the sample is weighted towards those who are older with a higher level of education. This means the findings cannot be generalised to populations who do not meet these demographics and they may have different motivators for BFP use which further research needs to explore. The survey was also self‐selecting meaning that although it shows variety in BFP use it is likely those with a high or low BFP use were most interested in taking part and should not be used to suggest these patterns are representative of the genuine population. From the responses, it appears that a subgroup of parents who followed BLW using snack BFP are included suggesting a different population group. However, this does give insight into how marketing and products may be affecting those who might not be typical BFP users previously.

We also used an online survey. Online research is now increasingly common, offering a wider diversity in sampling than when it first emerged, but still may be more accessible to those from more affluent backgrounds (Jang & Vorderstrasse, 2019). Our research was exploratory but further research may wish to consider how BFP are used and perceived by those on lower incomes.

We did not ask if infants were breastfed or what approach to introducing solid foods parents had taken–traditional weaning or BLW. This is a weakness and an opportunity missed to collect information about infant feeding in the UK.

Limitations aside the findings are important in understanding motivations for different BFP, product use and areas for supporting parent knowledge and education. They highlight how advertising and promotion of BFP, particularly snacks, are driving food choices and show the need for greater understanding of these tactics.

This research aimed to understand parents' perceptions of and motivators for using BFP or not. The findings show convenience is the main motivator for use. Specific products were chosen due to their perceived healthiness, despite caregivers not choosing BFP as they are the healthy option. This is due to health halo statements on packaging, although these are usually untrue. Moreover, caregivers who did not use BFP main motivators were distrust and preference to make their own. However, some alluded they would give BFP in the future, an indicator of how influential the introduction of baby snacks has been. Regulations and legislations around BFP must be tightened to help regulate the composition and labelling or products. Furthermore, those working with parents must be aware of these findings to help support with complementary feeding.

## AUTHOR CONTRIBUTIONS

Grace Hollinrake was responsible for study conception, study design, data collection, data analysis and draft report writing. Sophia Komninou was responsible for study design, supervision and draft report writing. Amy Brown was responsible for study conception, study design, data analysis support, supervision and draft report writing. All authors read and approved the final manuscript.

## CONFLICT OF INTEREST STATEMENT

The authors declare no conflict of interest.

## Supporting information

Supporting information.

## Data Availability

The data that support the findings of this study are available on request from the corresponding author. The data are not publicly available due to privacy or ethical restrictions.
