# Supplementary material for: Use of baby food products during the complementary feeding period: What factors drive parents' choice of products?
Source: Matern Child Nutr. 2024 Jun 19;20(4):e13689. doi: 10.1111/mcn.13689 (PMC11574642; doi:10.1111/mcn.13689)
Supplement: Supplementary file 1 — Supporting information. [file MCN-20-e13689-s001.docx]

**Appendix 1**

Questionnaire

Participant information

Q1 How old are you?

Q2 What is your sex?

Q3 What is your highest level of qualification?

- No formal qualification
- GCSE or equivalent
- A level or equivalent
- Degree level or equivalent
- Postgraduate or equivalent

Q4 What is your marital status?

- Married
- Cohabiting
- Single
- Divorced
- Widowed

Q5 What are the first 3 letters of your postcode?

Q6 Are you currently working?

- Yes full-time.
- Yes part-time.
- No

Q7 What is your ethnicity?

- White / White British
- Gypsy / Traveller
- Asian or Asian British: Pakistani
- Asian or Asian British: Bangladeshi
- Asian or Asian British Indian
- Asian or Asian British: Chinese
- Asian or Asian British: Other
- Black or Black British
- Mixed or multiple
- Irish
- Other
- Prefer not to say

Q8 How many children do you have?

Q9 How old is/are your baby/babies aged 4 – 12 months and what sex are they?

Commercial Baby Food and Snack Questions

Q10 How old was your baby when you first gave them solid foods?

Q11 Thinking about your baby’s diet, how often do you usually give them shop bought baby foods such as purees or baby snacks?

- More than once a day.
- Once a day
- Most days
- Once or twice a week
- Rarely
- Never

If never – please go to Q12 If never, what are your reasons for not using shop bought baby food products? – Final question.

If yes – please go to Q13 Which baby food brands do you use, and how often, please tick all that apply:

Q12 If never, what are your reasons for not using shop bought baby food products? __________

Q13 Why do you use shop bought baby food products?

|  | Strongly agree | Agree | Agree nor disagree | Disagree | Strongly disagree | Not applicable |
| --- | --- | --- | --- | --- | --- | --- |
| They are convenient |  |  |  |  |  |  |
| They save time |  |  |  |  |  |  |
| They are safe to feed my child |  |  |  |  |  |  |
| They are a healthier choice |  |  |  |  |  |  |
| My baby likes them |  |  |  |  |  |  |
| They are affordable |  |  |  |  |  |  |
| They are a good way to introduce new tastes and foods |  |  |  |  |  |  |
| My friends with babies use BFP |  |  |  |  |  |  |
| My family member recommended BFP |  |  |  |  |  |  |
| Healthcare Professional recommended BFP |  |  |  |  |  |  |
| They show what is suitable to feed at specific ages |  |  |  |  |  |  |
| Saw promoted on social media |  |  |  |  |  |  |
| Saw promoted in a baby magazine |  |  |  |  |  |  |
| I was given money off coupons |  |  |  |  |  |  |
| I used them with my other children |  |  |  |  |  |  |
| This is my first baby |  |  |  |  |  |  |

Q14 Which baby food brands do you use, and how often, please tick all that apply:

| Brand | More than once a day | Once a day | Every few days | Once a week | Once a month | Never | Not sure |
| --- | --- | --- | --- | --- | --- | --- | --- |
| Supermarkets own brand (e.g. Lupilu at Lidl, Mamia at Aldi, Sainsbury’s Little Ones, Asda Little Angels, Tesco) |  |  |  |  |  |  |  |
| Heinz |  |  |  |  |  |  |  |
| Ella’s kitchen |  |  |  |  |  |  |  |
| Cow & Gate |  |  |  |  |  |  |  |
| HiPP Organic |  |  |  |  |  |  |  |
| Little Freddie |  |  |  |  |  |  |  |
| Organix |  |  |  |  |  |  |  |
| Piccolo |  |  |  |  |  |  |  |
| For Aisha |  |  |  |  |  |  |  |
| Nutmeg |  |  |  |  |  |  |  |
| Kiddylicious |  |  |  |  |  |  |  |
| Babease |  |  |  |  |  |  |  |
| Little dish |  |  |  |  |  |  |  |
| Aptamil |  |  |  |  |  |  |  |
| Milupa |  |  |  |  |  |  |  |
| Cerelac |  |  |  |  |  |  |  |
| Tilda’s rice |  |  |  |  |  |  |  |
| Other |  |  |  |  |  |  |  |

If you use brand which is other please provide the name of the brand here: __________

Q15 How often does your baby have different types of shop bought baby food?

|  | More than once a day | Once a day | Every few days | Once a week | Once a month | Never | Not sure |
| --- | --- | --- | --- | --- | --- | --- | --- |
| Fruit only puree |  |  |  |  |  |  |  |
| Vegetable only puree |  |  |  |  |  |  |  |
| Fruit and vegetable mix puree |  |  |  |  |  |  |  |
| Fish based |  |  |  |  |  |  |  |
| Meat based |  |  |  |  |  |  |  |
| Desserts including yoghurt based |  |  |  |  |  |  |  |
| Cereals (e.g. baby rice, porridge, cerelac) |  |  |  |  |  |  |  |
| Baby crisps / puffs |  |  |  |  |  |  |  |
| Dried fruit snacks |  |  |  |  |  |  |  |
| Biscuits or cereal bars |  |  |  |  |  |  |  |
| Baby drinks |  |  |  |  |  |  |  |

Q16 If you give your baby jars or pouches of baby food, which do you use most regularly and why? __________

Q17 If you give your baby snack foods, which do you use most regularly and why? __________

The next question is about when choosing the specific brand and product.

Q18 Are any of these factors important to you when you choose baby food products for your child?

|  | Strongly agree | Agree | Agree nor disagree | Disagree | Strongly disagree |
| --- | --- | --- | --- | --- | --- |
| The composition is vegetarian |  |  |  |  |  |
| The product is halal certified |  |  |  |  |  |
| It is made from organic ingredients |  |  |  |  |  |
| It is gluten-free |  |  |  |  |  |
| It contains a variety of ingredients |  |  |  |  |  |
| They are full of fruits and vegetables |  |  |  |  |  |
| They include lots of different ingredients |  |  |  |  |  |
| They include nutritious / natural / real ingredients |  |  |  |  |  |
| It is a brand/type I can always buy easily |  |  |  |  |  |
| I trust this brand |  |  |  |  |  |
| It is a price I can afford |  |  |  |  |  |
| They are cost-effective compared to other brands |  |  |  |  |  |
| It is sold in my local shop |  |  |  |  |  |
| It is the most expensive |  |  |  |  |  |
| It is best quality |  |  |  |  |  |
| My baby likes them |  |  |  |  |  |
| Other people I know recommended them |  |  |  |  |  |
| It is the same I fed my other children (if applicable) |  |  |  |  |  |

Q19 Are there any other factors that are important to you when you choose baby food products for your child?

Q20 Can you explain why certain phrases / ingredients / approaches do or not increase the likelihood of you buying the product? __________

Q21 When it comes to feeding your child solid foods, how do you feel about the following?

|  | Very Confident | Confident | Confident nor Unconfident | Unconfident | Very unconfident | Not Applicable |
| --- | --- | --- | --- | --- | --- | --- |
| Deciding which brand to use |  |  |  |  |  |  |
| Knowing if the ingredients meet nutritional requirements |  |  |  |  |  |  |
| Deciding whether to buy sweet / savory meals and snacks |  |  |  |  |  |  |
| Combining commercial baby food with family foods |  |  |  |  |  |  |
| Knowing my child is getting enough food |  |  |  |  |  |  |
| Knowing the right portion sizes |  |  |  |  |  |  |
| Knowing how frequently my baby should eat |  |  |  |  |  |  |
| Knowing how to offer the food to my baby |  |  |  |  |  |  |
| How to store baby food once opened |  |  |  |  |  |  |
| How to warm baby food |  |  |  |  |  |  |
| Cooking my own food for my baby |  |  |  |  |  |  |

Q22 Is there anything else you are unsure about and feel you need more information about with regards to introducing solid foods to your child?
